# Erythème polymorphe secondaire à une infection à streptocoque alpha hémolytique au cours d'une maladie de Behҫet

**DOI:** 10.11604/pamj.2014.18.211.4891

**Published:** 2014-07-08

**Authors:** Wafa Chebbi, Olfa Berriche

**Affiliations:** 1 Service de Médecine Interne, CHU Taher Sfar Mahdia, 5100 Mahdia, Tunisie

**Keywords:** Erythème polymorphe, streptocoque alpha hémolytique, maladie de Behçet, Erythema multiforme, alpha-haemolytic streptococcus, Behcet disease

## Image en medicine

L’érythème polymorphe est un syndrome éruptif aigu, peu fréquent, caractérisé par l'aspect de des lésions cutanées, en cocarde. Il est le plus souvent associé à une infection à Herpes simplex virus ou à Mycoplasma pneumoniae. L'infection à streptocoque alpha hémolytique est exceptionnellement incriminée. Nous rapportons l'observation d'un homme âgé de 42 ans, suivi pour maladie de Behçet depuis deux ans, dont le diagnostic était retenu selon les critères internationaux (aphtose bipolaire, pseudofolliculites et pathergy test positif) et traité par colchicine (1mg/j). Il était hospitalisé pour une éruption érythémateuse maculopapuleuse bien limitée avec bordure foncée et centre clair en cocarde faisant 1 à 2 cm de grand axe siégeant au niveau du visage et au niveau des membres inférieurs et supérieurs, associée à une aphtose bipolaire, une fièvre à 40° C et des frissons. Il n'y avait pas de notion de prise médicamenteuse autre que la colchicine. La biopsie cutanée était en faveur d'un érythème polymorphe et montrait un épiderme largement nécrosé avec décollement sous-épidermique et une discrète atteinte des assises basales, un infiltrat dermique inflammatoire périvasculaire, polymorphe, lymphoplasmocytaire avec des polynucléaires neutrophiles. Les hémocultures étaient positives à streptocoque alpha hémolytique. Les sérologies du Mycoplasma pneumoniae, virus d'Epstein-Barr, cytomégalovirus, Herpes simplex virus 1 et 2, virus des hépatites B et C, virus de l'immunodéficience humaine, parvovirus B19, rougeole et de la fièvre Q étaient négatives. Une antibiothérapie associant le Céfapirine (Céfaloject^®^) à la dose de 6g/j et Gentamycine (160 mg/j) était instaurée. L’évolution était rapidement favorable avec disparition de la fièvre et régression complète des lésions cutanées.

**Figure 1:**
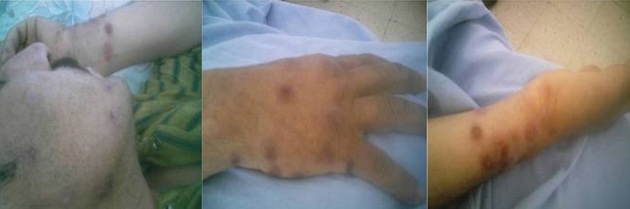
Eruption érythémateuse maculopapuleuse bien limitée avec bordure foncée et centre clair en cocarde siégeant au niveau du visage et au niveau des membres inférieurs et supérieurs

